# Methylation and gene expression patterns in adamantinomatous craniopharyngioma highlight a panel of genes associated with disease progression-free survival

**DOI:** 10.3389/fendo.2025.1585618

**Published:** 2025-06-12

**Authors:** Junier Marrero-Gutiérrez, Ana Carolina Bueno, Clarissa Silva Martins, Rui Milton Patrício Silva-Júnior, Antônio Carlos dos Santos, Marcelo Volpon Santos, Luiz Eduardo Wildemberg, Ximene Lima da Silva Antunes, Monica R. Gadelha, Ayrton Custodio Moreira, Ricardo Zorzetto Nicoliello Vêncio, Sonir Roberto R. Antonini, Margaret de Castro

**Affiliations:** ^1^ Department of Internal Medicine, Ribeirao Preto Medical School, University of São Paulo, Ribeirao Preto, Brazil; ^2^ Department of Pediatrics, Ribeirao Preto Medical School, University of São Paulo, Ribeirao Preto, Brazil; ^3^ Department of Medical Imaging, Hematology and Oncology, Ribeirao Preto Medical School, University of São Paulo, Ribeirao Preto, Brazil; ^4^ Surgery and Anatomy, Ribeirao Preto Medical School, University of São Paulo, Ribeirao Preto, Brazil; ^5^ Neuroendocrinology Research Center/Endocrinology Section, Medical School and Hospital Universitário Clementino Fraga Filho, Universidade Federal do Rio de Janeiro, Rio de Janeiro, Brazil; ^6^ Department of Computation and Mathematics Biology, Faculty of Philosophy, Sciences and Letters at Ribeirao Preto, University of São Paulo, Ribeirao Preto, Brazil

**Keywords:** transcriptome, molecular biomarker, progression-free survival, DNA methylation, adamantinomatous craniopharyngioma

## Abstract

**Introduction:**

Over the past decade, advancements in next-generation sequencing have significantly enhanced our understanding of the molecular pathogenesis of adamantinomatous craniopharyngiomas (ACP).

**Objective:**

This study integrated methylome and transcriptome analyses in ACP samples to explore the potential interplay between DNA methylation and RNA expression signatures for diagnostic and prognostic applications in ACP patients.

**Methods:**

This cross-sectional study evaluated clinicopathological features, DNA methylation, and gene expression profiles in 15 patients with ACP (33% women, age range: 3–55 years, 53% diagnosed before 18 years) treated at Ribeirao Preto Medical School, University of São Paulo.

**Results:**

Multidimensional scaling and principal component analysis identified two distinct clusters (ACP-A: n=9, ACP-B: n=6) with consistent composition across DNA methylation and gene expression profiles. While most clinical and histopathological characteristics were similar between clusters, ACP-A exhibited a longer median progression-free survival. ACP-B showed a higher prevalence of hypomethylated probes in CGI sites, and 63% of differentially methylated positions (DMPs) located in gene body regions. Differential methylation patterns were categorized into Methyl-Set1 (hypomethylated in ACP-A and hypermethylated in ACP-B) and Methyl-Set2 (hypermethylated in ACP-A and hypomethylated in ACP-B). Clustering analyses based on the methylation levels of probes and expression levels of the stringently filtered 212- and 37-gene sets further confirmed these two distinct ACP subgroups. Functional enrichment analysis highlighted key roles in synaptic modulation, nervous system development, cell adhesion, as well as pathways linked to RAS signaling, GTPase activity, and membrane potential regulation.

**Conclusion:**

Although clinical characteristics were largely comparable between the clusters, ACP-B patients exhibited shorter median progression-free survival, suggesting a more aggressive phenotype. The higher prevalence of hypomethylation in ACP-B indicates increased transcriptional activation, potentially driving tumor aggressiveness. The strong concordance between methylation and transcriptomic data in the 212- and 37-gene sets underscores their potential as a clinically relevant molecular biomarker panel. These gene sets demonstrate robustness in distinguishing ACP clusters, making it a promising tool for clinical sample classification.

## Introduction

Craniopharyngiomas (CPs) are rare intracranial neoplasms located mainly at the sellar and supra-sellar regions, along the anatomical developmental pathway of the craniopharyngeal duct with no clear gender preference (1–4). CPs are classified into two main subtypes: adamantinomatous (ACP) and papillary (PCP). While both subtypes share characteristics such as anatomical location, expression of adult pituitary stem cell markers, glial reaction proteins, and cytokeratin, they differ in clinical, morphology, histological features, epidemiology, and biological behavior (5, 6). They also exhibit distinct epigenetic and molecular profiles, suggesting they are biologically different entities (7–11).

Over the last decade with the development of next generation sequencing methods, advancements on molecular pathogenesis of CPs have been made, including the discovery of the BRAF p.V600E mutation in the majority of PCPs. This mutation enhances MAPK/ERK signaling, increasing the proliferative capacity of SOX2+ cells. This prevents pituitary differentiation into hormone-producing pituitary cells, resulting in cell transformation, tumor formation and growth (10, 12, 13). In contrast, based on murine models, a different mechanism is hypothesized for the development of ACPs. Gain-of-function mutations in the *CTNNB1* gene, present in most ACPs, lead to the activation of the WNT/beta-catenin pathway, which triggers SOX2-expressing cell clusters to promote the proliferation and invasion of neighboring tissues (14–17).

Due to their proximity to critical structures such as the optic nerve, third ventricle, hypothalamus, pituitary stalk, and internal carotid artery and its branches, CPs are associated with significant morbidity. This includes hypopituitarism, hypothalamic dysfunction, hypothalamic obesity, visual and neurological deficits, and cognitive impairments (5, 18, 19). Subtotal resection of CPs is linked to higher recurrence rates, making adjuvant radiation therapy often necessary (20). These conditions contribute to the lowest quality of life reported among patients with pediatric and adult brain tumors (18, 21, 22), including severe obesity (23).

Transcriptional profiling using microarrays or RNA sequencing has underscored the importance of inflammatory, odontogenic, and MAPK/ERK pathways in ACP growth and invasiveness. The role of the MAPK/ERK pathway has also been demonstrated in pediatric ACPs through proteomic data, suggesting potential therapeutic targets (24–26). A comprehensive bioinformatic analysis of publicly data identified hub genes that might serve as genetic markers of diagnosis, treatment, and prognosis of ACP, confirming the involvement of chemical synaptic transmission, cell adhesion, odontogenesis of the dentin-containing tooth, cell junction, extracellular region, extracellular space, structural molecule activity, and structural constituent of cytoskeleton (27).

More recently, we conducted a comprehensive analysis of ACP methylation data, identifying two distinct methylation signature clusters. In this study, unsupervised hierarchical cluster analysis (UHCA) revealed a significantly hypomethylated cluster enriched with *CTNNB1*-mutated ACPs, which was associated with increased tumor size. Gene enrichment analyses of both clusters highlighted pathways described above involving mainly tumor proliferation and the tumor microenvironment (28). We also showed that *CTNNB*1-mutated ACPs have shorter telomeres, demonstrating a relationship between the Wnt/β-catenin pathway and telomere biology in the pathogenesis of these tumors (29). In another previous study, we showed that unsupervised analysis of integrated methylome and transcriptome signatures was capable of classifying either functional pituitary tumors, such as somatotrophinomas and corticotrophinomas, or non-functional pituitary tumors. These signatures were associated with clinical presentation and also with tumor invasiveness, which impacts the management of the disease (30).

Common limitations in such studies include methodological heterogeneity and reliance on publicly available cohort datasets. Current state of genomic findings reinforces the importance of integrating methylome and transcriptome analyses in ACPs. Here, we addressed these issues by using a well-characterized subset of ACP samples from a Brazilian cohort. This approach allowed us to combine methylome and transcriptome analyses on the same samples, with the aim of uncovering insights into the potential integration of DNA methylation and RNA expression signatures for diagnostic and prognostic applications in ACP patients.

## Materials and methods

### Patients and biological specimens

This study was conducted in accordance with ethical guidelines, including the Declaration of Helsinki, and received approval from the Ethics Committee of the University Hospital at Ribeirao Preto Medical School, University of São Paulo (FMRP-USP; approval #7534/2010). Written informed consent was obtained from all patients or their legal guardians.

In this cross-sectional study, we evaluated 15 subjects with clinically diagnosed and pathologically confirmed ACP, followed at FMRP-USP (n = 8, with data on endocrine and clinical manifestations) or at the Federal University of Rio de Janeiro (UFRJ, n = 7), both in Brazil. These subjects represent a subset of a larger cohort in which the methylation profile of ACP samples was recently analyzed (28). In the current study, additional molecular analyses focusing on the transcriptome profile of these samples were conducted. Demographic, clinical, biochemical, and outcome data were collected from the patients’ medical records. The clinical diagnosis of ACP was established following a comprehensive clinical assessment and laboratory testing, as recommended (19). Hypopituitarism was diagnosed in accordance with international guidelines (31), and obesity was identified using the body mass index (BMI) z-score (32). Data on radiological findings, treatment, and outcomes were collected for all patients. Surgical procedures were performed by the respective neurosurgical teams. Tumor progression was defined as recurrence after total resection or an increase in the size of a residual lesion requiring reoperation. Total resection was confirmed when no residual tissue was detected, whereas partial resection was defined by the presence of remaining tumor tissue on postoperative imaging. All patients underwent ophthalmologic evaluations and imaging via magnetic resonance imaging (MRI) and/or computed tomography (CT). Lesion size was measured along its largest axis, and tumor volume (cm³) was calculated using the formula width × height × length × 0.5 (33). Pre- and postoperative MRI and CT scans were analyzed by a single neuroradiologist (ACS). Hypothalamic invasion was assessed according to Puget et al. (34). Histopathological diagnosis included the presence of ACP featured characteristics, such as peripheral palisading of epithelial cells; central stellate reticulum-like cells; nodules of “wet keratin” (anucleate, eosinophilic material); calcifications; cystic areas containing cholesterol-rich, brownish fluid; reactive gliosis, often accompanied by Rosenthal fibers, and chronic inflammation in surrounding tissue. The samples were also tested for the presence of *CTNNB1* activating mutations.

### Nucleic acid extraction

DNA and RNA were extracted from microdissected, fresh frozen ACP samples using QIAamp DNA Mini Kit (QIAGEN, Hilden, Germany) and RNeasy kit (QIAGEN, Hilden, Germany), respectively, according to the manufacturers’ instructions. Quantification was performed by fluorometry using Qubit dsDNA BR and Qubit RNA BR Assays (Qubit Fluorometer, Thermo Fisher Scientific, Waltham, MA). Integrity was assessed by electrophoresis using the TapeStation 4200 System (Agilent, Santa Clara, CA). We considered adequate DNA integrity number (DIN) ≥6, and RNA integrity number (RIN) ≥7.

### Molecular profiling analyses

#### Methylome profiling analysis

DNA methylation analysis was commissioned by the University of Southern California Keck Genomics Platform (USC-KCP, Los Angeles, CA, USA) using the Infinium Methylation EPIC BeadChip Array (Illumina, San Diego, CA, USA). Raw intensity files provided by the facility were then processed through a standard quality control pipeline established by our team, as previously described (28). The *minfi* R package (v1.30.0) was used for data preprocessing and to report methylation levels as *M*-values [log_2_(methylated/unmethylated)] (35, 36). Bias correction was performed using the preprocessQuantile function (37). Data filtering was applied to remove probes with a detection *P*-value greater than 0.01, probes located on sex chromosomes, probes containing single nucleotide polymorphisms at CpG sites, and cross-reactive probes (38). The genomic annotation of the CpG sites was assessed according to Illumina’s manifest file version 0.3.0. Their localization relative to gene sub-regions was categorized as TSS1500 and TSS200 (1,500 and 200 bp upstream transcription star sites, respectively), and 5’UTR (5’ untranslated region, between TSS and the ATG start site) — which comprehended the gene’s TRR; the gene’s first exon; body (between the ATG and stop codon); exon boundary, and 3’UTR (between the stop codon and poly A signal). DMPs localization relative to CpG islands (CGIs) was categorized as Shore - up to 2 kb upstream (N) and downstream (S) of the CGI, Shelf - 2–4 kb upstream (N) and downstream (S) of the CGI, and Open Sea.

In addition, we submitted our methylation raw data files (.IDATs) to the Heidelberg EpiGnostix platform (CNS Tumor Methylation Classifier v12.8) (39), which enables robust epigenetic comparison against central nervous system (CNS) tumor profiles, as defined by the latest World Health Organization guidelines. This analysis classifies samples as matching to a defined DNA methylation class (“match”: scores ≥ 0.9), or “no match” (scores < 0.9) which is still relevant for cases with low tumor content or poor DNA quality, being scores > 0.3 relevant only for superfamily matching prediction (39).

Methylation data are publicly available in the NCBI’s Gene Expression Omnibus (GEO) under accession number GSE239695 (40).

#### Transcriptome profiling analysis

RNAseq analysis of ACP was also commissioned to the USC-KGP. Briefly, total RNA was used in ribosomal RNA (rRNA) depletion (NEB E6310), which was subsequently fragmented to a target size of 180 bp by heat fragmentation. RNA library preparation, cDNA conversion and polyA selection were performed using the NEBNext^®^ Ultra™ II Directional RNA Library Prep Kit for Illumina (E7765, New England Biolabs) according to the manufacturer’s instructions. Each library was normalized and pooled the same day of sequencing. The 101*101 paired-end sequencing was performed on Illumina’s NovaSeq 6000, using the NovaSeq S4–200 cycles flow cell (Illumina, San Diego, CA) V1 chemistry. All sequencing reads were converted to industry standard FASTQ files using BCL2FASTQ v1.8.4, and provided by the facility. The FASTQ files were merged and aligned to the human genome version GRCh38 using STAR v2.5.3a (40). Duplicate reads in the aligned BAM files were identified using Picard Mark Duplicates (v1.128). Counting reads for the genomic features was obtained with featureCounts (v1.6.0) (41). We applied variance-stabilizing transformation (VST) to the read counts for downstream analysis (42). Transcriptome data are publicly available in the NCBI’s GEO under accession number GSE294056 (43).

#### Unsupervised clustering analyses

Multiple unsupervised approaches were employed independently in each dataset (Methyl- and Transcript-datasets) to cluster ACP samples. In Methyl-dataset analysis, we employed post-quality control *M*-values. In Transcript-dataset analysis, we counted matrix read-counts after VST. For both datasets, we conducted multidimensional scaling (MDS) analysis using Euclidean distance as the dissimilarity metric to visualize the results, and performed a principal component analysis (PCA). The procedures were implemented using the packages “*stats*” (44), “*Pvclust*” (45), and “*FactoMineR*” (46).

#### Methylation and differential gene expression analyses

In order to identify the differentially methylated probes (DMPs) between the clusters obtained by the Methyl-dataset analysis — ACP-A and ACP-B —, we followed our previously described pipeline (40). Briefly, the median probes’ *M*-values were compared between the clusters using the Mann-Whitney U test and the Benjamini-Hochberg method to adjust the false discovery rate (*P*_adj_), and considering DMPs those with *P*_adj_ ≤ 0.05. After annotating the DMPs according to the Illumina’s B4 v1.0 manifest, we categorized them based on their relative location in CpG islands (CGIs) and genetic subregions. We next evaluated the association of these categories with cluster formation using the chi-square test. Finally, the most informative DMPs were selected, namely: (i) Methyl-Set1, those hypomethylated in all ACP-A samples and hypermethylated in all ACP-B samples (*M*-values ACP-A < 0 < *M*-values ACP-B); and (ii) Methyl-Set2, those hypermethylated in all ACP-A samples and hypomethylated in all ACP-B samples (*M*-values ACP-A > 0 > *M*-values ACP-B).

In order to evaluate differentially expressed genes (DEGs) between the clusters obtained by the Transcript-dataset analysis — ACP-A and ACP-B — we used the DESeq2 package (47). We set the significance threshold for differential expression at a *P*_adj_ ≤ 0.01 and an absolute value of log_2_ fold-change (FC) ≥ 2. We chose to employ rigorous criteria when assessing changes in gene expression fold differences between the two ACP clusters.

#### Correlation between methylation and gene expression

We examined whether differences in median methylation in probes overall and transcriptional regulatory regions (TRRs) among clusters corresponded to the expression patterns of the genes they regulate. To explore the correlation between methylation and gene expression, we accomplished two distinct approaches. Approach I: focus on methylation dynamics by correlating the *M*-values of the probes within Methyl-Set1 and Methyl-Set2 with the matrix reads of the respectively queried genes. Then, we distinguished the subset of probes targeting the genes’ TRRs, estimated the median genes’ *M*-values and, subsequently, correlated them with the respective genes’ expression values. Approach II: focus on gene expression dynamics by correlating the matrix reads of the DEGs within clusters with their corresponding targeted methylation probes. We identified TRR-related probes, calculated their median *M*-values, and performed correlation analyses, evaluating the expression of a gene regulated by the methylation of its promoter. In addition, we narrowed down this approach by considering only the genes whose probes were within Methyl-Set1 or Methyl-Set2. We employed the Pearson correlation coefficient (*r*) for all correlation calculations and considered values with *r* ≤ -0.7 or *r* ≥ 0.7 and *P*_adj_ ≤ 0.05 as statistically significant. These calculations were implemented using the “Hmisc” package in R (48).

#### Enrichment analysis

We performed Gene Ontology (GO) enrichment analysis for biological processes of methylation data, taking into consideration two distinct sources of bias: the number of DMPs per gene and DMPs annotated across multiple genes. We employed the “*gometh*” function from the *missMethyl* Bioconductor package (49) selecting terms exhibiting over-representation with a significance threshold of *P* ≤ 0.05. Furthermore, we conducted GO enrichment analyses using the relevant gene-sets identified in each approach of our analyses (P ≤ 0.05) using the “*enrichGO*” function from the *clusterProfiler* package (50).

#### Statistical analysis

Continuous or discrete variables were reported individually, collapsed (mean and median) or as percentage, as informed in figure legends and tables. The association with statistically significant categorical variables distribution between groups was calculated using the chi-square test. Differences of continuous variable distribution between groups were calculated using the Mann Whitney-U test. Progression-free survivals (PFS) was analyzed using Kaplan–Meier curves and was defined as the time elapsed from diagnosis until the last follow-up, considering metastasis/recurrence as unfavorable events. Patients who were lost to follow-up were censored considering their last follow-up visit. Log-rank test was used for the comparison of survival rates between groups. The minimum level of statistical significance was set at *P*-value ≤ 0.05, but more stringent options are also presented when appropriate. All analyses were implemented in R statistical language (version 4.3.0) (44).

## Results

### Patients and clinical presentation

**Table 1** summarizes the demographic, clinical, and molecular features of the patients with ACPs. Amongst the 15 patients with ACP, 33% were women. Based on self-reported skin color, 53% declared to be brown, 40% white, and 7% black. The median age at diagnosis was 15 years [range: 3 – 55], being 53% of the patients diagnosed before 18 years of age. At baseline – time of the first surgical procedure – among those with clinical and endocrine data available, 50% were overweight or obese, and we observed neurological [headache (89%), visual field alteration (75%), and convulsive seizure (25%)], hypothalamic (vomiting (33%), no hyperphagia nor hypersomnia), and pituitary deficiency findings (hypopituitarism in 64%, being at least one hormone deficiency in 67% and 33% presented with two deficiencies). Only 22% of the patients presented AVP deficiency at diagnosis. All tumors evaluated presented calcified lesions (100%), and most were solid-cystic (92%). The tumors’ median size was 2.8 cm (1 - 3.4), and the median volume 9.8 cm^3^ (3 - 38.6). Regarding hypothalamic involvement, most of the tumors - 79% - were classified as type 2, 14% as type 1, and 7% with no invasion (type 0).

**Table 1:** Demographic, clinical, histopathological, and molecular features of patients with adamantinomatous craniopharyngioma (ACP) at baseline and according to the molecular clusters.

Features	All ACP	ACP-A	ACP-B	*P-value* ACP-A vs ACP-B
Demographic				
Patients, n (%)	15 (100)	9 (60)	6 (40)	–
Reference center, n (%)				
FMRP-USP	8 (53)	4 (44)	4 (67)	0.60
UFRJ	7 (47)	5 (56)	2 (33)	
Sex, n (%)				
Men	10 (67)	8 (89)	2 (20)	0.09
Women	5 (33)	1 (11)	4 (80)	
Age at diagnosis (years)				
Mean	20.7	21.2	20	0.67
Median	15	12	17	
Range	3 - 55	3 - 55	7 - 49	
< 18	8 (53)	5 (56)	3 (50)	0.83
18 – 60	7 (47)	4 (44)	3 (50)	
Self-reported skin color, n (%)				
Brown	8 (53)	3 (33)	5 (83)	0.16
White	6 (40)	5 (56)	1 (17)	
Black	1 (7)	1 (11)	0 (0)	
Endocrine and clinical manifestations *				
Time interval between the appearance of signs and symptoms and the diagnosis (months)				
Mean	19.1	13	27.3	0.68
Median	18	15	18	
Range	1 – 60	1 – 21	4 – 60	
Time interval between the diagnosis and the initial surgical treatment (months)				
Mean	16.7	5.4	33.7	0.86
Median	5.1	5.1	4.3	
Range	0.3 – 176.2	0.3 – 10.9	0.7 – 176.2	
Hypopituitarism, n (%)				
Present	7 (48)	5 (46)	2 (33.3)	>0.99‡
Absent	4 (26)	2 (27)	2 (33.3)	
Not available	4 (26)	2 (27)	2 (33.3)	
Pituitary hormone deficiencies - per axis**, n (%)				
ACTH	0	0	0	0.81
GH	4 (22)	3 (33)	1 (11)	
LH/FSH	2 (11)	1 (11)	1 (11)	
TSH	1 (6)	0	1 (11)	
AVP	2 (11)	1 (11)	1 (11)	
None	1 (6)	0	1 (11)	
Not available	8 (44)	4 (45)	4 (45)	
Body mass index classification***				
Underweight, n (%)	1 (7)	0	1 (17)	0.66
Healthy weight, n (%)	3 (20)	1 (11)	2 (33)	
Overweight, n (%)	2 (13)	2 (22)	0	
Obesity, n (%)	2 (13)	1 (11)	1 (17)	
Not available	7 (47)	5 (56)	2 (33)	
Nonspecific symptoms of increased intracranial pressure, n (%)				
Headache, n (%)				
Present	8 (53)	4 (44.5)	4 (67)	0.34‡
Absent	1 (7)	1 (11)	0	
Not available	6 (40)	4 (44.5)	2 (33.3)	
Nausea, n (%)				
Present	3 (20)	1 (11)	2 (33.3)	0.52‡
Absent	6 (40)	4 (44.5)	2 (33.3)	
Not available	6 (40)	4 (44.5)	2 (33.3)	
Convulsive seizures, n (%)				
Present	2 (13)	0	2 (33)	0.03‡
Absent	6 (40)	5 (56)	1 (17)	
Not available	7 (47)	4 (44)	3 (50)	
Visual impairment, n (%)				
Present	9 (60)	5 (56)	4 (67)	>0.99‡
Absent	3 (20)	2 (22)	1 (16.5)	
Not available	3 (20)	2 (22)	1 (16.5)	
Primary lesion features				
Size (cm)				
Mean	2.4	2.2	2.5	0.74
Median	2.7	2.3	2.7	
Range	1– 3.4	1 – 3.2	1.2 – 3.4	
Volume (cm^3^)				
Mean	13.4	13	13.5	0.43
Median	9.8	9.9	6.4	
Range	3 – 38.1	8 – 26.7	3 – 38.6	
Macroscopic classification, n (%)				
Solid	1 (7)	1 (11)	0	0.99‡
Solid-cystic	12 (80)	6 (67)	6 (100)	
Not available	2 (13)	2 (22)	0	
Hypothalamic invasion				
Type 0	1 (7)	1 (11)	0	0.47‡
Type 1	2 (13)	2 (22)	0	
Type 2	11 (73)	5 (56)	6 (100)	
Not available	1 (7)	1 (11)	0	
Primary lesion – treatment features****				
Surgical approach, n (%)				
Transcranial	12 (80)	7 (78)	5 (83)	0.99‡
Transsphenoidal	2 (13)	1 (11)	1 (17)	
Not available	1 (7)	1 (11)	0	
Resection, n (%)				
Total	2 (13)	2 (22)	0	0.48
Partial	13 (87)	7 (78)	6 (100)	
Tumor progression, n (%)				
Present	9 (60)	5 (56)	4 (66)	>0.99‡
Absent	4 (27)	3 (33)	1 (17)	
Not available	2 (13)	1 (11)	1 (17)	
Radiotherapy throughout follow-up, n (%)				
Present	9 (60)	5 (56)	2 (33)	0.61
Absent	8 (40)	4 (44)	4 (67)	
Follow-up (years)				
Mean	105.2	127	72.5	0.22
Median	60	72.6	44.4	
Range	18 - 437	18.4 - 418	18 – 210.1	
Outcome, n (%)				
Alive	14 (93)	8 (89)	6 (100)	> 0.9
Deceased	1 (7)	1 (11)	0 (0)	
Somatic *CTNNB1* genotype, n (%)				
Wild type	3 (20)	1 (11)	2 (33.3)	0.2‡
Mutant	10 (67)	8 (89)	2 (33.3)	
Not evaluated	2 (13)	0	2 (33.3)	

^‡^Patients whose data were unavailable, or those who were lost to follow-up, were not included in the analyses.

*Endocrine and clinical manifestations data are available only for patients from FMRP-USP.

**Some ACP patients present with more than one pituitary hormone deficiency.

***Body mass index was classified according to age and sex percentiles in patients younger than 20 years at diagnosis (CDC) or according to WHO in patients older than 20 years at diagnosis.

****Therapeutic approach consisted of surgical resection of primary-naïve lesions for radiotherapy, chemotherapy or local immunotherapy followed by radiotherapy after the initial surgery or during follow-up.

Differences between the variables in the groups were assessed using chi-square or Fisher’s exact tests (discrete), and t test or Mann-Whitney test (continuous).

All patients underwent surgical treatment. Transcranial approach was more frequent than transsphenoidal (86 vs 14%). Partial tumor resection occurred in 87% of the patients. One surgery was performed in 6 (40%) patients, whereas 7 (47%) underwent two surgeries, and 2 (13%) more than two surgeries. Recurrence occurred in 69% of the patients; median time to tumor progression was 31 months (4 – 148), and 7 (47%) received adjuvant radiotherapy. Only one patient (7%) died, as a result of infection after surgical procedure. From available data on the clinical outcomes, we observed increasing frequency of hormone deficiencies during the follow-up after treatments (median: 60.2 months, range: 18 - 437): AVP (22 vs 73%), LH/FSH (22 vs 53%), GH (44 vs 53%), TSH (11 vs 73%), and ACTH (0 vs 73%). The number of patients with at least one affected axis increased after treatment from 89 to 100%. Hypersomnia (0 vs 12.5%) and hyperphagia (0 vs 62.5%) also increased, as well obesity (25 vs 75%).

These data obtained from the current subset of patients with methylome and transcriptome data were similar with data obtained from the entire cohort (28).

#### Molecular profiling analyses

**Figure 1:**
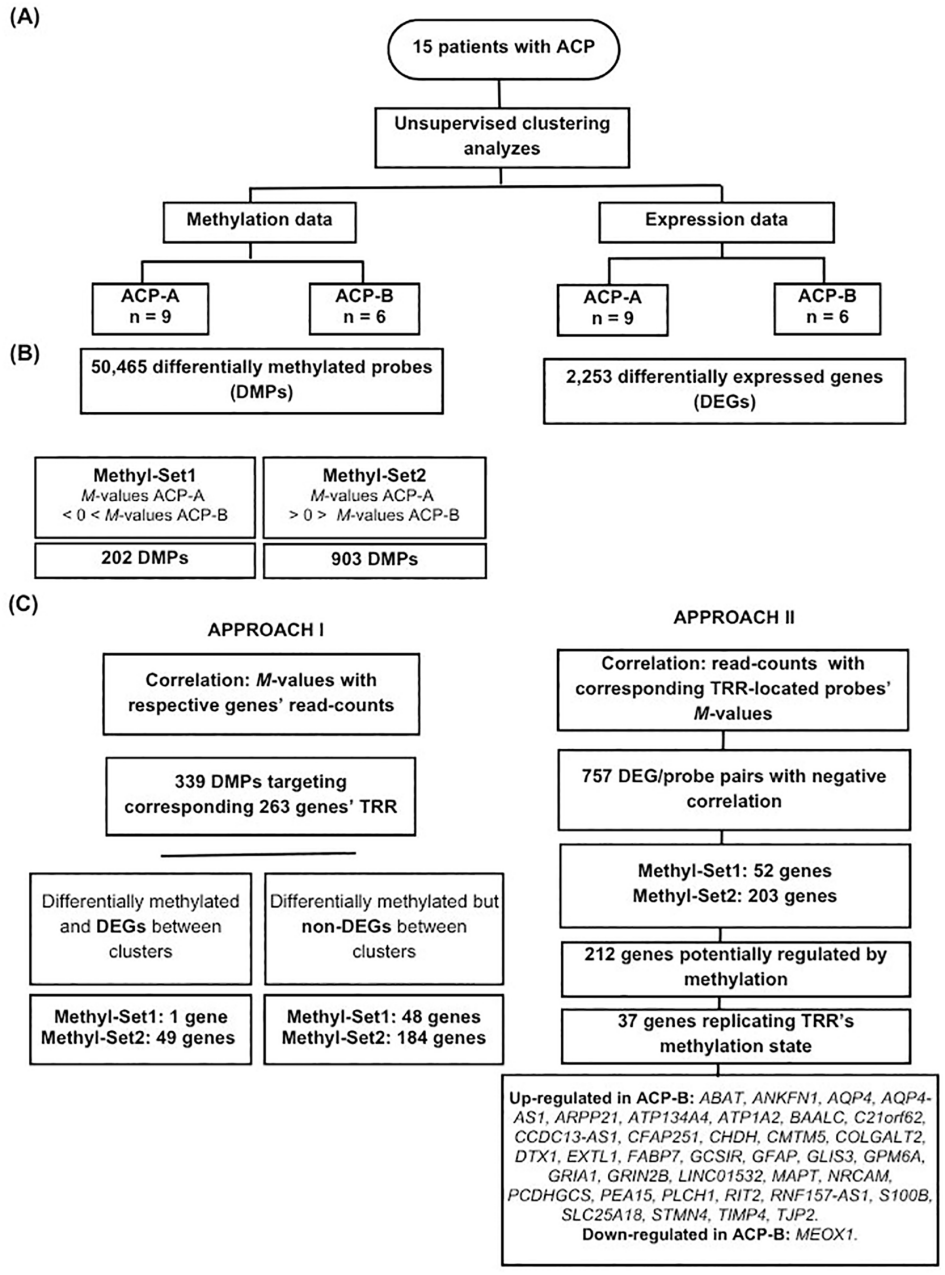
Summary of methylation and transcriptome profiling analyses flowchart and the main findings of the study. **(A)** Unsupervised clustering analyzes from the tumor samples from 15 patients with adamantinomatous craniopharyngiomas (ACPs) were performed using multidimensional scaling (MDS) and principal component analysis (PCA) considering the methylation (M-values) and expression (read-counts) values. **(B)** Identification of differentially methylated probes (DMPs) and differentially expressed genes (DEGs) between the clusters. **(C)** Integrating methylation and expression data using Pearsons’ correlation analyses. Approach I: focus on methylation dynamics by correlating the M-values of the probes within Methyl-Set1 and Methyl-Set2 with the matrix reads of the respectively queried genes. Approach II: focus on gene expression dynamics by correlating the matrix reads of the DEGs within clusters with their corresponding targeted methylation probes.

In order to illustrated and summarize the studies of ACP’s methylation (Approach I) and transcriptome (Approach II) profiling analyses, a flowchart is presented in **Figure 1**.

#### Methylation and gene expression profiles yield the same clustering of ACP samples

**Figure 2:**
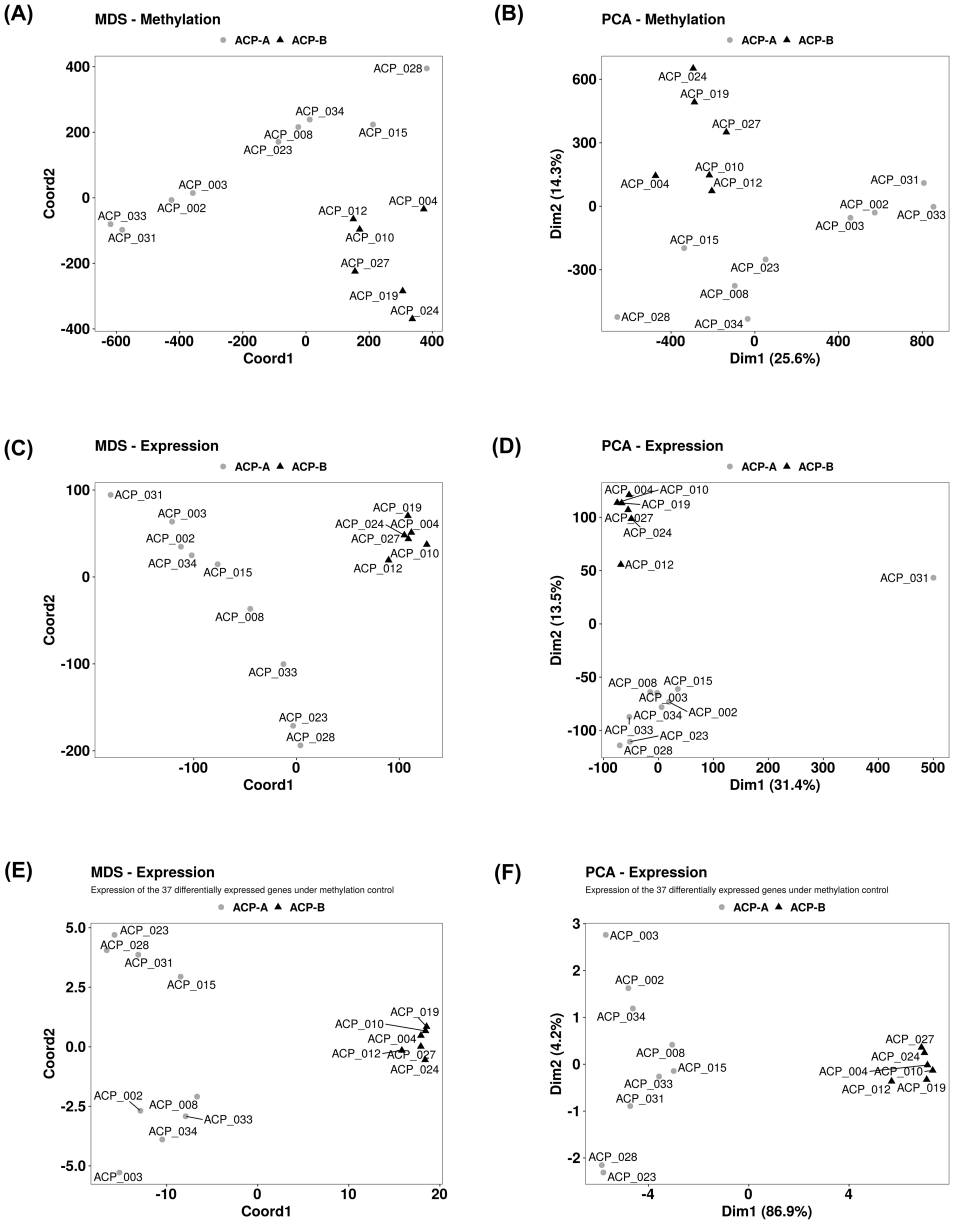
Unsupervised clustering of tumor samples from 15 patients with adamantinomatous craniopharyngiomas (ACPs) according to methylation and gene expression data independently supported the existence two well defined clusters with the same sample composition: ACP-A (gray circles) and ACP-B (black triangles). Two analyses were applied: multidimensional scaling (MDS) and principal component analysis (PCA). **(A, B)** Methylation values (M-values) from all 772,531 eligible methylation probes. **(C, D)** Expression values (read-counts matrix applied to the variance stabilizing transformation) from 61,860 genes. **(E, F)** Methylation and expression values from a panel of 37 genes whose transcription regulating regions (TRR) methylation state were strongly and significantly correlated with their expression levels (list presented in **Figure 1**).

**Figure 3:**
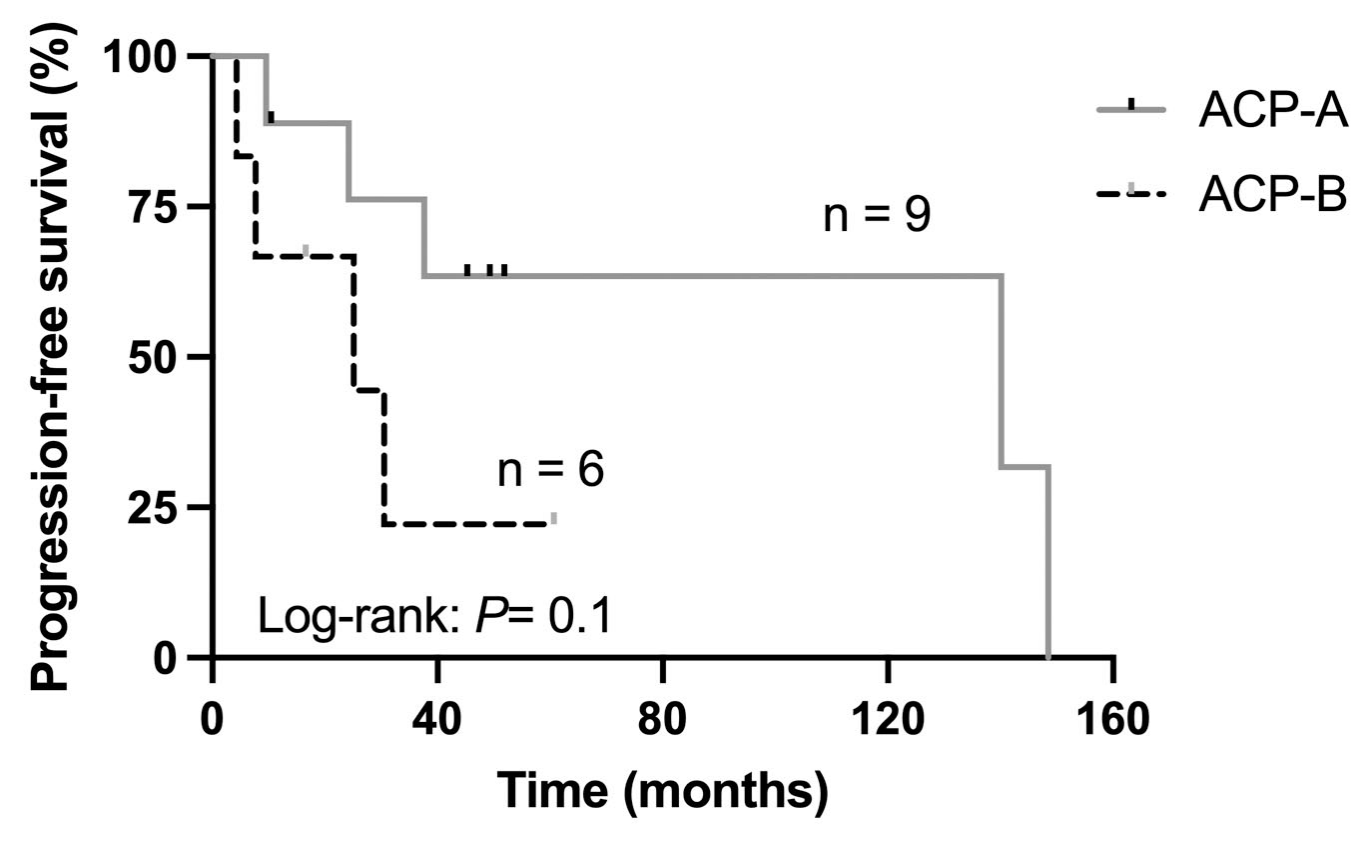
Kaplan-Meier plot presenting the disease progression-free survival of 15 patients with adamantinomatous craniopharyngiomas (ACP) according to the clustering resulted from DNA methylation and gene expression profiling.

Methyl-dataset was composed by 772,531 probes filtered in after the quality control step [available at reference (40)], whereas Transcript-dataset consisted of 61,860 genes annotated into reference genome GRCh38 (**Supplementary Table S1**). The multidimensional scaling (MDS) analysis performed using both datasets from the ACP samples individually rendered two well defined clusters. Interestingly, the composition of the clusters for both DNA methylation and gene expression profiles contained the same ACP samples, and were named ACP-A (n = 9) and ACP-B (n = 6) (**Figures 2A, C**). PCA analyses conducted with these datasets also confirmed the existence of two clusters with the same composition of ACP samples (**Figures 2B, D**). With exception of higher incidence of convulsive seizures in patients from cluster ACP-B, most demographic, clinical, histopathological, neuro-ophthalmological, and radiological characteristics of ACP patients at baseline did not differ in the clusters. Throughout the follow-up, there were no differences in the frequencies of tumor progression (62% and 80%, *P* > 0.9), in of patients submitted to more than one surgical proceeding (55 vs 67%, *P* = 0.67), nor submitted to adjuvant radiotherapy (56% vs 33%, *P* = 0.61). However, even considering the small sample size, the median disease PFS tended to be higher in subjects from cluster ACP-A than from ACP-B (140.1 vs 25.1 months; *P* = 0.1, **Figure 3**).

The EpiGnostix analyses demonstrated that samples from cluster ACP-A (ACP_002, ACP_003, ACP_008, ACP_015, and ACP_023) were characterized as typical of midline tumors, particularly CPs. Samples ACP_002 and ACP_023 presented matching classification scores (≥ 0.9) confirming canonical methylation signature associated with CPs, whereas samples ACP_003 and ACP_008, even exhibiting not matching classifier scores (0.6 and 0.8, respectively), were also predicted to CP family. Notably, ACP_015, predicted to be a benign meningioma (score 0.93), was also included in the cluster ACP-A. All tumors in Cluster A were *MGMT* promoter unmethylated. Cluster ACP-B included samples ACP_004, ACP_010, ACP_012, ACP_019, and ACP_024. Samples ACP_004 (score 0.94) and ACP_012 (score 0.4) matched with reactive microenvironmental tissue family, which likely represent samples with low tumor cellularity and predominance of stromal or inflammatory glial elements. ACP_010 yielded a non-informative result (score <0.3), suggesting possible DNA degradation. Samples ACP_019 and ACP_024 (both scores >0.9) corresponded to low-grade glial or glioneuronal tumors. Only ACP_019 exhibited *MGMT* promoter methylation across cluster ACP-B.

#### Differential methylation and expression analysis between ACP clusters

**Figure 4:**
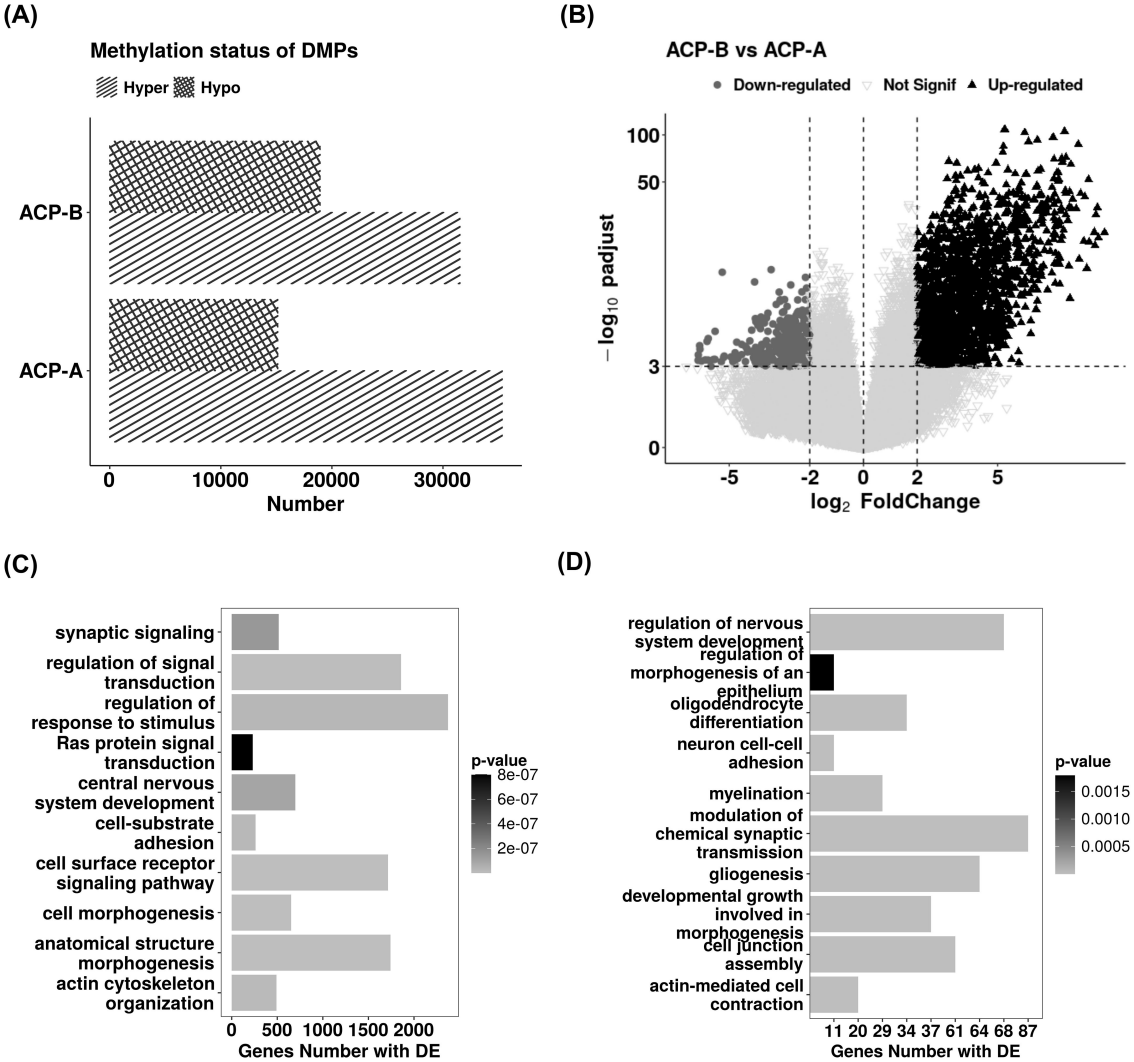
Analysis of differential DNA methylation and gene expression between the clusters ACP-A and ACP-B. **(A)** Distribution of differentially methylated probes status between the clusters. **(B)** Volcano plot showing the differential gene expression between the clusters ACP-A and ACP-B (median expression difference Padj ≤ 0.01 and an absolute value of log2). **(C, D)** Enriched biological processes from genes with differentially methylated probes and with differential expression, respectively.

A total of 50,465 probes were differentially methylated between the clusters ACP-A and ACP-B (**Supplementary Table S2**). ACP-B had a greater number of hypomethylated probes (18,936 probes vs 15,130 in ACP-A), while ACP-A had the highest number of hypermethylated probes (35,335 probes vs 31,528 in ACP-B) (**Figure 4A**). In both clusters, CGI sites were hypomethylated; however, ACP-B maintained the lowest methylation status of CGI. Hypermethylation was a feature of non-CGI sites and globally. Considering the DMPs distribution according to CpG island location, we observed that 69% are situated in the OpenSea. Both the Shelf-N and Shore-N, as well as the Shelf-S, Shore-S regions, exhibited a significant association with the methylation status of the clusters (chi-square test *P*≤ 0.05; **Supplementary Table S3**), with ACP-B displaying the greatest hypomethylation (**Supplementary Figure S1**). The same was observed when considering the DMPs location in the genes sub-regions (1stExon, 3’UTR, 5’UTR, Body, ExonBnd, TSS1500, TSS200): hypomethylation in ACP-B and hypermethylation in ACP-A (**Supplementary Figure S2**). Of note, most DMPs (63%) were found in the genes’ body region. The analysis of the differential gene expression between the clusters revealed 2,253 DEGs (**Supplementary Table S4**): 1,850 were up-regulated and 403 were down-regulated in ACP-B compared to ACP-A (**Figure 4B**).

We observed that processes such as synaptic modulation, nervous system development (glial cell differentiation, gliogenesis, learning, behavior), response to stimuli, and cell adhesion are enriched in both, methylation and transcriptome levels (**Figures 4C, D**).

#### The impact of DNA methylation on gene expression regulation

We observed that 75% of the DMPs share the same methylation state - either hypomethylated or hypermethylated - across both clusters (**Supplementary Figure S1**). Therefore, we refined our analysis to focus on the 1,105 DMPs that highlight differences in methylation status between the clusters, referred to as Methyl-Set1 (n = 202; hypomethylated in all ACP-A samples and hypermethylated in all ACP-B samples) and Methyl-Set2 (n = 903; hypermethylated in ACP-A samples and hypomethylated in all ACP-B samples) (Approach I; **Figure 1C** left panel; **Supplementary Table S5**). Within these Methyl-Sets, we identified 339 probes annotated to genes’ transcriptional regulatory regions (TRRs) distributed as follows: 27% targeting TSS1500, 15% targeting TSS200, 49% targeting the 5’UTR, and 9% targeting the 1st Exon. These TRR-associated probes were annotated to 263 unique genes (**Supplementary Table S6**).

In order to evaluate the influence of DNA methylation on the gene expression, we examined the overlap between genes with differential TRR methylation and those showing differential expression between the clusters. Amongst the 49 genes identified from Methyl-Set1, only one hypomethylated gene (corresponding to 2%), the *NACC2*, was also differentially expressed, but interestingly, underexpressed. For the 234 genes represented by the probes in Methyl-Set2, 50 genes overlapped with DEGs: 49 genes exhibited the typical pattern between methylation and expression (hypomethylation in the TRR in Methyl-2 and upregulation in ACP-B; **Supplementary Table S7**) with an exception of the *PRRX2* gene, which displayed an atypical pattern of methylation and gene expression. We also evaluated the correlation between the *M*-values and the expression levels of the residual probes from the Methyl-Sets which overlapped with non-DEGs: 48 genes from Methyl-Set1 and 184 from Methyl-Set2 (**Supplementary Table S7**). This approach rendered a total of 27 genes whose methylation levels were strong and significantly correlated with their expression levels. Amongst these genes, 10 exhibited whole TRR methylation behavior akin to that of individual probes, like the *ZBTB18* gene, for example, whose TRR was hypomethylated and negatively correlated with its expression levels (**Supplementary Figure S3**).

**Figure 5:**
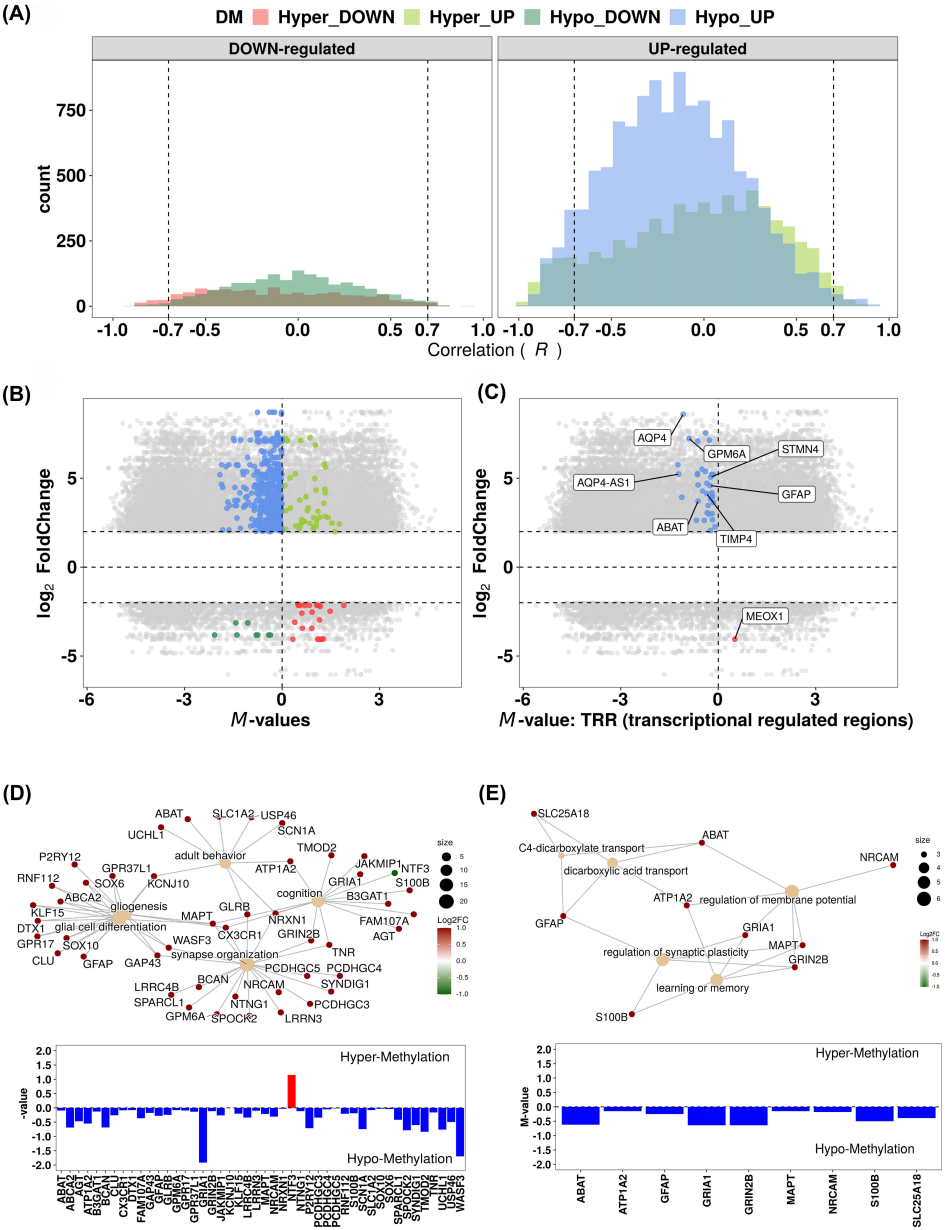
Analysis of the methylation profile of differentially expressed genes (DEGs) between the clusters and identification of a gene panel of clinical interest resulting from the approach II described in the manuscript and summarized in **Figure 1**. **(A)** Histogram showing the correlation between the methylation levels (M-values) of probes and expression values (read-counts) of their respective annotated DEGs. Highlighted are the relationships between probe methylation levels and expression, with ACP-B as reference. Hyper_Down: hypermethylated probe/downregulated gene Hyper_Up: hypermethylated probe/upregulated gene. Hypo_Down: Hypomethylated probe/downregulated gene. Hypo_Up: hypomethylated probe/upregulated gene. **(B, C)** Scatter plots of gene expression (log2 fold-change) and mean methylation difference. Each point represents a CpG-gene pair (n = 212; **B**) or transcription regulatory region (TRR) methylation-gene pair (n = 37; **C**), emphasizing pairs with a strong and significant Pearson’s correlation. **(D, E)** Enriched biological processes from gene-sets composed by the 212 CpG-gene and 37 TRR methylation-gene pairs, respectively.

We next evaluated whether the 2,253 DEGs between ACP-B and ACP-A (**Supplementary Table S4**) were influenced by their own promoter methylation (Approach II, **Figure 1C** right panel). Firstly, we calculated the correlation between the *M*-values and the expression values of 21,927 probe/DEG pairs (**Supplementary Table S8A**). Among these, 1,639 (7.5%) probe/DEG pairs were significantly correlated, being 1,416 (86.4%) negatively correlated: 1,338 hypomethylated probes/up-regulated genes and 78 hypermethylated probes/downregulated genes in ACP-B; and 223 (13,6%) were positively correlated: 35 hypomethylated probes/downregulated genes and 188 hypermethylated probes/up-regulated genes (**Figure 5A**). We next narrowed down our analysis by selecting only the probe/DEGs pairs whose probes had different methylation states between clusters: those from Methyl-Set1 or Methyl-Set2. We identified 67 probes from Methyl-Set1 interrogating 52 DEGs (41 up- and 11 downregulated) and 416 probes from Methyl-Set2 interrogating 203 DEGs (201 up- and 2 downregulated), yielding a total of 255 genes. Among these genes, a set of 212 (84%) genes were strongly and significantly correlated with their TRR methylation levels/status between clusters: 24 hypermethylated DMPs representing 11 down-DEGs and 408 hypomethylated DMPs associated with 201 up-DEGs (**Figure 5B**, **Supplementary Table S8B**). Further narrowing down the analysis, we searched within these 212 gene-set for those that replicated the methylation state of their entire TRR, and we identified 37 genes (36 upregulated and 1 downregulated: *MEOX1*; **Figures 1**, **5C**, **Supplementary Table S8C**). Among these, we found some key genes previously reported to be involved in tumor invasion and progression in other types of tumors, such as *GPM6A, ABAT, GFAP*, and *AQP4*.

Interestingly and of clinical interest, we replicated the clustering analyses (MDS and PCA) using the methylation levels of the probes annotated to 212- and 37-gene sets, and their expression values. Again, either for methylation or for gene expression, we observed two well defined clusters with the same composition of ACP samples using all the data generated by methylated and transcriptome profiling (**Figures 2E, F**).

When studying the enriched biological processes in the genes targeted by DMPs, as well as those obtained from differential expression analyses (**Supplementary Tables S9A-C**), we observed that the same processes such as synaptic modulation, nervous system development - glial cell differentiation, gliogenesis, learning, behavior, response to stimuli, and cell adhesion - were also enriched when considering the genes composing the 212- and 37- gene sets. In addition, we also identified genes belonging to the protein RAS and GTPase signaling transduction, dicarboxylic acid transport, and membrane potential regulation (**Figures 5D, E**).

## Discussion

In this study, we analyzed the transcriptome of a subset of ACP samples for which the methylation profile had been previously characterized (28), integrating DNA methylation with RNA signatures. The demographic, clinical, biochemical, imaging, treatment, and outcome data from these patients closely matched the data from our overall cohort (28) and the literature (5, 29). Unsupervised analyses of methylation and gene expression profiles in ACP samples revealed two distinct clusters (ACP-A and ACP-B), with consistent grouping across both datasets, as confirmed by MDS and PCA analyses. Considering that both groups consisted of ACP samples and given the inherently unpredictable nature of these tumors, as well as concerns about the small sample size, further investigation is required to fully understand the clinical implications of these clusters’ classification. Importantly, we would like to emphasize that, although some important between-clusters differences did not fulfill statistical significance, they may still be clinically relevant, and may indicate an important trend that deserves to be explored.

To investigate cluster-specific differences, we identified differentially methylated probes (DMPs) and differentially expressed genes (DEGs) between them. Both clusters exhibited hypomethylation at CpG island (CGI) sites, with ACP-B showing the lowest levels in CGI sites and overall. In mammals, the inhibition or activation of transcription by methylation is dependent on the gene segment analyzed: methylation in the promoter is inversely correlated with the expression, whereas methylation in the gene body is positively correlated with expression (51). As a hallmark of neoplasms development, global tumor DNA hypomethylation increases as a lesion progresses from a benign proliferation of cells to an invasive cancer, and it is associated with a higher transcriptional activation (52, 53). In this sense, the ACP-B hypomethylation profile suggests a more aggressive biological behavior, which is in line with the tendency of lower PFS observed in the patients in this cluster. Indeed, 63% of DMPs found in our study were annotated to gene body regions and were also hypomethylated in ACP-B. The same pattern was observed in OpenSea regions and nearby shores and shelves. These regions are typically linked to gene silencing and the modulation of regulatory environments, influencing chromatin structure and transcriptional activity, highlighting the importance of non-CGI methylation patterns in genomic stability and transcriptional control (53).

Since in our study, 75% of DMPs maintained the same methylation state across both clusters, we analyzed the most informative probes from Methyl-Set1 and Methyl-Set2, what revealed distinct methylation patterns. We focused on DMPs linked to TRRs, which regulate transcription initiation (TSS1500 and TSS200), translation and mRNA stability (5’ UTR), and gene expression (1st Exon). These probes were associated with unique genes, suggesting that methylation at these sites may influence gene expression and impact biological processes in both clusters (53, 54). In Methyl-Set1, only one hypomethylated gene, *NACC2* (2%), deviated from the typical pattern where hypomethylation enhances gene expression, suggesting that *NACC2* may be regulated by additional epigenetic mechanisms. *NACC2* encodes a protein that acts by protein-protein association functioning as a transcriptional regulator, controlling the expression of genes involved in neural development and differentiation, and in developmental signals, potentially as a tumor suppression (55). In Methyl-Set2, 50 genes overlapped with DEGs, mostly following the expected hypomethylation-upregulation pattern, except for *PRRX2* (Paired Related Homeobox 2 gene), a transcription factor crucial for embryonic development and mesodermal differentiation (56). Inhibition of *PRRX2* was previously correlated with lower expression of genes controlling epithelial-mesenchymal transition, a process linked to cancer progression (57), specially through inactivation of Wnt/β-catenin pathway (58) — a hallmark of ACP. The finding that such an important gene as *PRRX2*, hypomethylated and downregulated in ACP_B, suggest that gene expression may be influenced by alternative regulatory mechanisms beyond DNA methylation. Correlation analysis, which has been extensively utilized to explore the relationship between methylation and gene expression (59, 60), revealed genes whose methylation levels strongly correlated with their expression, even without differential expression between clusters. Ten genes, including *ZBTB18*, exhibited consistent TRR hypomethylation pattern linked to increased expression. This suggests that although these genes might not exhibit differential expression between the clusters they may still be regulated by methylation or other mechanisms like miRNA interactions, which can act alongside or against DNA methylation in gene regulation (59, 61, 62). *ZBTB18* plays a crucial role in brain development, neural progenitor cells differentiation, cell proliferation, and migration (59, 62). In these contexts, *ZBTB18* expression regulates several pathways in glioblastoma (63), which are also active in ACP microenvironment and clearly related to tumor development.

Correlation analysis also identified 212 genes potentially regulated by promoter methylation, with 37 genes (36 upregulated, 1 downregulated) showing consistent TRR methylation patterns. Clustering analyses based on methylation and expression levels of these 212- and 37-gene sets replicated the same composition of ACP samples in ACP-A and ACP-B clusters, when using all the data generated by the entire data from methylated and transcriptome, reinforcing their biological relevance. Notably, this gene set could serve as a clinically valuable biomarker panel to distinguish ACP patient groups regarding disease PFS (ACP-A: 140.1 vs. ACP-B: 25.1 months). The observed consistency across both methylation and transcriptomic data underscores the robustness of this gene-set as a marker for segregating clinical samples into these distinct ACP clusters.

Within the 37-gene-set, we highlight *GPM6A* (Glycoprotein M6A), *ABAT* (4-Aminobutyrate Aminotransferase), *GFAP* (Glial Fibrillary Acidic Protein), and *AQP4* (Aquaporin-4), known to play roles in tumor invasion and progression within various cancers. *GPM6A* is a membrane glycoprotein primarily involved in neuronal development and plasticity. Recent studies suggest its role in cancer progression, particularly in brain tumors, by influencing cell adhesion, migration, and invasion. It may contribute to glioma and other neural-origin cancers by affecting neural-like pathways in tumor cells (64). *ABAT* encodes an enzyme responsible for GABA catabolism at the mitochondrial matrix level. It plays an essential role in the nucleoside salvage pathway and its deficiency leads to neurometabolic disorder and loss of mitochondrial DNA copy number (65). Decreased *ABAT* gene expression, including due to hypermethylation, was shown to be associated with aggressive tumor behavior, resistance to adjuvant chemotherapy, and poor prognosis in some primary non-CNS cancers, such as breast, liver and adrenocortical carcinomas (66–68). On the other hand, *ABAT* appears to play a tumor-promoting role in certain metastatic settings, particularly those involving the nervous system, such as disseminated medulloblastoma and breast cancer brain metastases, suggesting that these tumors can utilize ABAT to exploit neuronal GABA as a readily available energy source (69). In line, our data showed that *ABAT* is not repressed by methylation, but it is in fact hypomethylated throughout its entire TRR and overexpressed in ACP samples from cluster ACP-B, what could render mitochondrial metabolic advantages for tumor proliferation and disease recurrence in these patients. *GFAP* is key intermediate filament protein in astrocytes, used as a marker for gliomas and astrocytomas. It plays a role in maintaining the integrity of the glial network but is also implicated in glioblastoma progression. GFAP-positive gliomas are known for their aggressive and invasive nature (70). AQP4 is a water channel protein primarily found in the brain and involved in maintaining fluid homeostasis. In glioblastomas and other brain tumors, AQP4 has been linked to increased edema, tumor cell migration, and invasion. Targeting AQP4 has been explored as a potential strategy for reducing tumor-associated swelling and spread in malignant gliomas (71). Therefore, each of these genes has significant roles in cancers, particularly in gliomas, and now our data also suggest their role in ACP, where they might contribute to invasion, metabolic shifts, and tumor microenvironment changes. Thus, a gene panel, including these genes, holds promise as a predictive tool for patient outcomes in clinical settings. However, further large-scale studies are necessary to validate these findings and ensure their clinical reliability.

Functional analysis of methylome data revealed enriched processes such as synaptic modulation, nervous system development, glial cell differentiation, gliogenesis, learning, behavior, and cell adhesion, which were also identified in gene expression analysis. These processes are critical for neuronal communication, synaptic plasticity, and tumor microenvironment dynamics (72). Additional enriched pathways included RAS and GTPase signaling, dicarboxylic acid transport, and membrane potential regulation, suggesting that hypomethylation may enhance gene expression and optimize signaling and energy metabolism (73). Notably, RAS and GTPase pathways showed increased activity in ACP-B, potentially contributing to its phenotype. A recent study analyzing public datasets supports these findings, highlighting genes involved in synaptic transmission, cell adhesion, extracellular matrix, and cytoskeletal structure cytoskeleton (27).

DNA methylation-based classification of CNS tumors such as EpiGnostix is usually used to increase diagnostic confidence and precision, especially when histopathology is unclear (39). Although it was not our case, once the histology and the molecular findings of our samples were compatible with ACPs, this method enriched our tumor grouping findings. Cluster ACP-A encompassed epigenetically well-defined profile of samples, including benign origin tumors such as CPs and meningioma. Despite its distinct biological origin, meningioma may share partial epigenetic features with CPs, such as low molecular aggressiveness, and epigenetic dysregulation of WNT-related genes, endorsing its inclusion in ACP-A. On the other, EpiGnostix recognized ACP-B as a more heterogeneous cluster of samples, with inflammatory reactive tissue and low-grade glial tumors and *MGMT* status variability.

In line with ACP heterogeneity, recent advances in scRNA-seq, snRNA-seq, and spatial transcriptomics have highlighted significant inter- and intra-tumoral heterogeneity, revealing diverse cell populations, including epithelial, immune, and stromal cells (74). A recent study identified six ACP tumor cell subsets based on *CTNNB1* mutation status. Among them, the whorl-like cluster exhibited distinct molecular features with activated WNT/β-catenin and SHH signaling. Palisading epithelium cells contained a proliferating subset, while another subpopulation expressed high cytokine levels and SASP factors. Some cells showed elevated mitochondrial gene expression, and certain ACPs had clonally expanded cytotoxic T cells. Additionally, two novel subpopulations—senescent and germinal cells—were identified, each with distinct molecular and morphological characteristics (75). Another recent study using snRNA-seq and spatial transcriptomics found that the ACP environment is immunosuppressive, with tumor-associated macrophages (TAMs) highly infiltrating the microenvironment. The study identified a regulatory network facilitating RHCG+ epithelial cell keratinization, contributing to tumor progression (76). TAMs and cancer-associated fibroblasts (CAFs) may drive immune suppression and tumor growth via AXL signaling. Further analysis revealed that palisade-like, basaloid-like, and whorl-like epithelium cells in ACPs express AXL, which activates signaling pathways promoting proliferation, survival, adhesion, migration, and invasion (77). Targeting senescent cells with senolytic agents or inhibiting AXL receptors (e.g., with Bemcentinib) could be promising strategies for ACP treatment as observed in various epithelial malignancies (76, 77).

These studies utilizing scRNA-seq, snRNA-seq, and spatial transcriptomics may highlight some apparent limitations of our work. However, there remains significant value in studies employing whole-tumor transcriptomics. This approach involves simpler analytical pipelines compared to the computationally intensive workflows required for single-cell and spatial data, thereby reducing the risk of errors and misinterpretations. Its simplicity makes it particularly well-suited for hypothesis generation, large-scale studies, and initial screening, especially in clinical or resource-constrained settings. Ultimately, while single-cell and spatial transcriptomics reveal previously inaccessible layers of complexity in ACP pathogenesis, whole-tumor transcriptomics provides complementary strengths in scalability, reproducibility, and practicality. Together, these methodologies provide a comprehensive and synergistic framework for advancing our understanding of ACP biology and improving future therapeutic strategies.

## Conclusion

Overall, our data offer detailed insights into the dynamics of methylation and gene expression in ACP, shedding light on key regulatory mechanisms and identifying potential prognostic biomarkers. The identification of distinct ACP clusters based on methylation and transcriptomic profiles paves the way for personalized treatment strategies and the development of clinically relevant prognostic panels.

## Data Availability

The datasets presented in this study can be found in online repositories. The names of the repository/repositories and accession number(s) can be found below: https://www.ncbi.nlm.nih.gov/, GSE239695 and GSE294056.
